# Multipollutant, longitudinal analysis of the association between urinary tungsten and incident diabetes in a rural population

**DOI:** 10.1097/EE9.0000000000000173

**Published:** 2021-10-13

**Authors:** Emily Riseberg, Katherine A. James, Mark Woodin, Rachel Melamed, Tanya Alderete, Laura Corlin

**Affiliations:** a Department of Public Health and Community Medicine, Tufts University School of Medicine, Boston, Massachusetts; b Department of Environmental and Occupational Health, Colorado School of Public Health, University of Colorado-Anschutz Medical Campus, Aurora, Colorado; c Department of Civil and Environmental Engineering, Tufts University School of Engineering, Medford, Massachusetts; d Biological Sciences, University of Massachusetts, Lowell, Lowell, Massachusetts; e Department of Integrative Physiology, University of Colorado, Boulder, Colorado.

## Abstract

**Background:**

Cross-sectional studies suggest tungsten (W) exposure may be associated with diabetes. We assessed longitudinal associations between urinary W and fasting glucose, 2-hour glucose, insulin resistance (HOMA-IR), β-cell function (HOMA-β), and incident type 2 diabetes.

**Methods:**

We used data from 1,609 Hispanic and non-Hispanic White adults with 20 to 74 years of age residing in rural Colorado and participating in the San Luis Valley Diabetes Study. Urinary metal exposure values were measured at baseline and natural log-transformed. We assessed longitudinal associations between urinary W and continuous outcome measures using linear-mixed effect models and associations with incident diabetes using Fine and Gray competing risks regression models (competing event = all-cause mortality). The main adjustment set of covariates included: age, sex, ethnicity, education, smoking status, hypertension, body mass index, caloric intake, alcohol intake, and urinary creatinine levels. Secondary models were further adjusted for arsenic, cadmium, and lead exposures. We assessed whether sex or ethnicity were effect modifiers.

**Results:**

At baseline, the median W concentration was 0.22 μg/L (interquartile range = 0.20, 0.59). In the main cross-sectional analyses, lnW levels were significantly associated with 3% higher lnHOMA-IR (95% CI = 1 to 5). In the main longitudinal models, lnW was significantly associated with 1% higher natural log-transformed fasting glucose (95% CI = <1 to 1), 3% higher natural log-transformed HOMA-IR (95% CI = 2 to 5), and 28% higher incident diabetes (subdistribution hazard ratio=1.28, 95% CI = 1.09 to 1.50). Results remained significant when further adjusting for other metals. We observed evidence for effect modification by sex and ethnicity.

**Conclusion:**

Urinary W was longitudinally associated with adverse metabolic health indicators.

## Abstract 2

Supplemental Digital Content is available in the text.

**What this study adds**

We evaluated longitudinal associations between urinary tungsten and fasting glucose levels, 2-hour glucose, insulin resistance (HOMA-IR), β-cell function (HOMA-β), and incident diabetes using data from the San Luis Valley Diabetes Study, a biethnic cohort from rural Colorado. After adjustment for covariates and urinary arsenic, cadmium, and lead exposures, tungsten was significantly associated with fasting glucose, HOMA-IR, and incident diabetes. Given that tungsten has industrial applications and exposure disparities persist, our results could inform future diabetes prevention efforts.

## Introduction

In the United States, 34.1 million adults (13% of the adult population) have diabetes mellitus, and approximately 90 to 95% of cases are type 2 diabetes (hereafter, diabetes).^1^ Although the prevalence is high among most subpopulations, diabetes disproportionately affects individuals residing in rural areas.^2,3^ Diabetes is a complex disorder that develops as a result of both genetic and environmental factors.^4^ For example, toxicological and epidemiologic studies suggest that exposure to certain metals (e.g., arsenic, cadmium, and lead) may be associated with diabetes and impaired fasting glucose levels^5,6^ through mechanisms including oxidative stress and inflammation, impaired glucose metabolism, and impaired insulin secretion and storage.^7–9^

Tungsten (W) is a transitional metal found naturally in soil, water, food, air, and particulate matter.^10,11^ The use of W in industrial settings has been increasing due to its high melting point, flexibility, and strength.^10,12^ W is also frequently found in household items and medical supplies.^13^ Currently, there are no federal guidelines on the levels of W in drinking water,^14^ but W has been listed as an emerging metal of concern by the Environmental Protection Agency and the National Toxicology Program.^15,16^ Individuals who work with W or who live in areas with high levels of W in the water may have elevated urinary W compared with the general population.^11^ W has been associated with increased incidence of lung cancer, high blood pressure, stroke, and chronic kidney disease.^10,17–19^ Cross-sectional studies conducted in the United States and China suggest that urinary W is also positively associated with fasting glucose and diabetes prevalence.^6,20^ However, no cross-sectional studies have examined associations between W and certain other markers of diabetes (e.g., β-cell function), and no longitudinal studies of which we are aware have examined the association between W and any continuous biomarker of diabetes.

Additionally, although W may potentiate adverse health effects of exposure to metals such as cobalt and nickel,^10^ no studies have examined how relationships between W and diabetes may be affected by coexposure to other elements. One study among US women found that overall exposure to metal mixtures was associated with diabetes incidence, but W was not included in the mixture analyses due to a low percentage of participants with detectable measurements.^21^ Finally, despite known sex differences in the risk factors for and pathophysiology of diabetes,^22–24^ and despite structural racism that results in differential exposure to diabetes risk factors by ethnicity,^25^ no previous studies examined whether sex or ethnicity modify associations between W and diabetes.

We sought to address these gaps in the literature using data from the prospective San Luis Valley Diabetes Study (SLVDS). This study population is of particular concern since the W levels in the water sources are elevated due to depleting water supply.^19,26,27^ Specifically, our *a priori* primary objective was to assess whether urinary W levels were longitudinally associated with fasting glucose levels, 2-hour glucose levels, insulin resistance, β-cell function, and diabetes incidence. Our *a priori* secondary objectives were to assess whether these associations were robust when adjusting for coexposure to other metals and to assess whether sex or ethnicity modified the associations.

## Methods

### Study population

The San Luis Valley is a rural region of Colorado covering over 8,000 square miles in six counties (including Alamosa and Conejos counties) with a total population of about 46,000 residents.^28^ Approximately 49% of the people in the region identify as Hispanic.^28^

### Study sample

**Figure 1.**
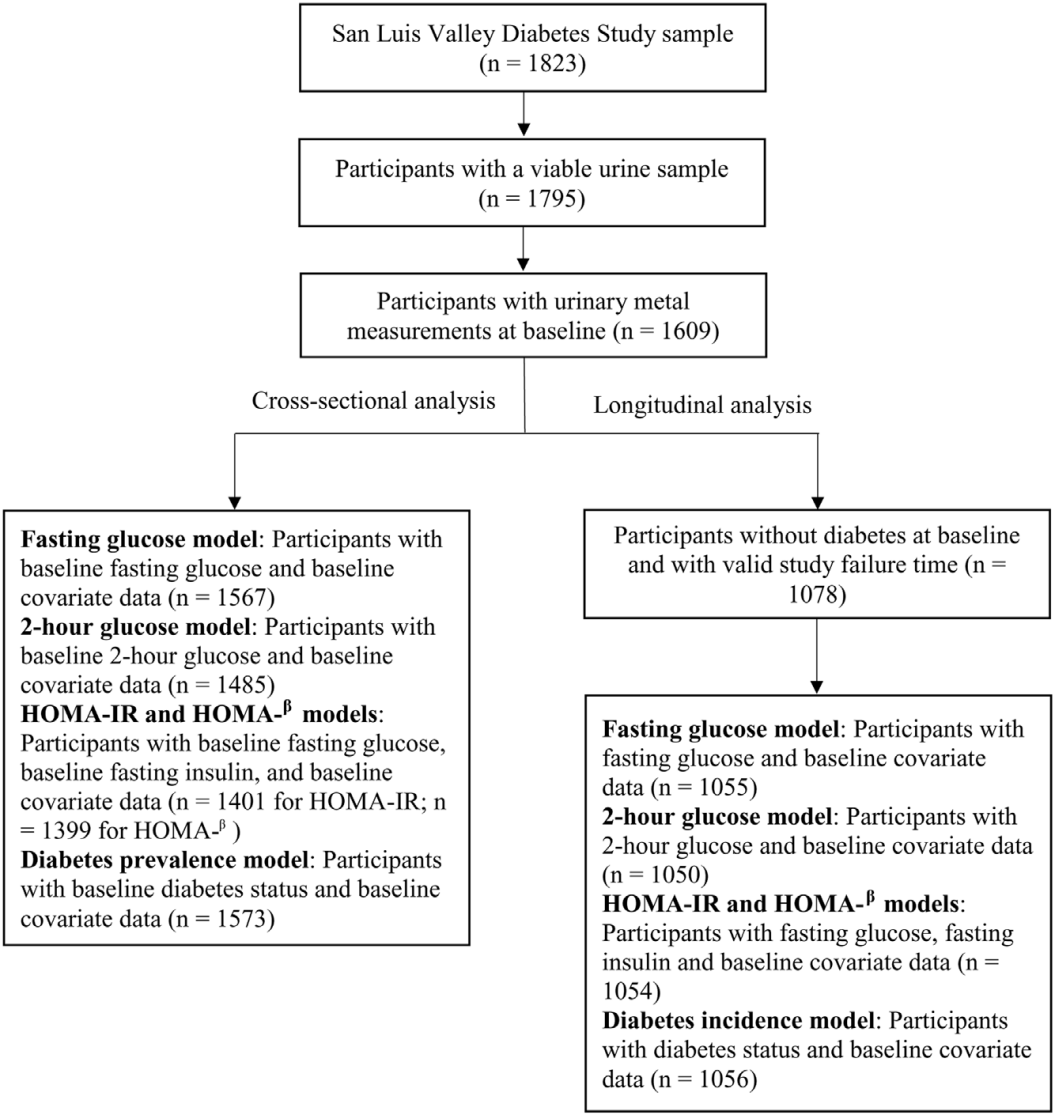
Analysis sample selection scheme.

Participant recruitment and data collection methods for the SLVDS have been described previously.^29^ Briefly, the SLVDS was designed to investigate risk factors for diabetes and other chronic diseases among Hispanic and non-Hispanic White adults. The primary identification stage of recruitment differed for diabetics and nondiabetics, and recruitment in each category included two phases with the same recruitment protocols (phase 1: 1984–1985; phase 2: 1986–1987). Individuals with a previous diagnosis of diabetes were identified and recruited through medical records and advertisements (n = 479 attended the first examination cycle, 343 of whom were identified in phase 1). Individuals who were not previously known to have diabetes were identified using a two-stage geographic sampling procedure. The first stage used maps, directories, and other information to sample approximately 21% of households in Alamosa and Conejos counties. In the second stage, individuals were selected at random within county, sex, age, and ethnic group strata to reflect the Hispanic population with diabetes in the study area (n = 1,344 attended the first examination cycle, 607 of whom were identified in phase 1). Regardless of diabetes status, individuals were eligible to enroll in the study if they were (1) 20–74 years of age, (2) current residents of either Alamosa or Conejos counties, (3) able to complete the interview in English or Spanish, and (4) able to give informed consent. All 1,823 SLVDS participants who were in the original study were eligible for this study if they had urinary metal measurements at their first (baseline) examination cycle (n = 1,609, of whom 373 were considered to have diabetes and 1,237 were not; see Figure 1 for sample selection).

### Data collection

The first (baseline) examination cycle occurred between 1984 and 1988, with follow-up examination cycles occurring over 10 years. At each examination cycle, researchers obtained demographic, clinical, and behavioral data. Participants reported their age, sex, ethnicity (Hispanic/non-Hispanic), educational attainment (<12 years/12 years/>12 years), and smoking status (never smoker [<100 lifetime cigarettes]/former smoker/current smoker). Blood pressure was measured three times at each examination cycle, and the average of the second and third measurements were used. Participants were considered to have hypertension if their average systolic blood pressure was ≥140 mmHg, their average diastolic blood pressure ≥90 mmHg, or if they currently used blood pressure-lowering medication.^30^ Height and weight measured at the examination were used to calculate body mass index (BMI; kg/m^2^; obesity defined as BMI ≥ 30 kg/m^2^). A food frequency questionnaire was used to calculate the participants’ total caloric intake per day and alcohol intake per week.^31^

Urine samples (approximately 120 ml) were collected in trace metal-free tubes at baseline examination cycles between 1984 and 1988. Samples were stored in a freezer at –80°C until they were analyzed in 2008 and 2015 at two laboratories as part of separate studies. In 2008 (n = 529) and 2015 (n = 504), the Colorado Department of Public Health and Environment chemistry laboratory analyzed a full metals panel (including W, antimony, arsenic, barium, cadmium, cesium, chromium, cobalt, copper, lead, manganese, molybdenum, plutonium, selenium, thallium, uranium, and zinc) on 1,033 urine samples. In 2015, Columbia University Metals Laboratory analyzed a full metals panel for an additional 576 samples. Both laboratories met the standards of the Clinical Laboratory Improvement Amendment. They used an inductively coupled argon plasma instrument with a mass spectrometer to detect metal concentrations (detection limit for each metal = 1 part in 10)^32^ and a colorimetric assay by the Jaffe reaction to assess urinary creatinine levels.^33^ For urine samples with metal concentrations below the level of detection, concentrations were imputed using the square root of the limit of detection divided by two.

Outcome measures (fasting glucose, 2-hour glucose, insulin resistance, β-cell function, and diabetes status) were assessed at each examination cycle. Participants fasted for at least eight hours before the examination. Blood was drawn once before consuming glucose through a flavored drink and again at 1 and 2 hours after consumption to collect data on glucose and insulin levels. Participants with diabetes using insulin did not inject the insulin until after the first blood sample was collected, and participants taking oral hypoglycemic medication took the medication before the examination. Glucose was measured using the glucose oxidase method with venous plasma,^29,34^ and insulin was measured with a double antibody radioimmunoassay.^35,36^ Homeostatic Model Assessment of Insulin Resistance (HOMA-IR) was calculated as the product of fasting insulin (μU/mL; to convert to pmol/L, multiply by 0.144) and fasting glucose (mg/dL; to convert to mmol/L, multiply by 0.056) divided by 405. Homeostatic Model Assessment of beta-cell function (HOMA-β) was calculated as [20 times fasting insulin (μU/mL)]/[fasting glucose (mg/dL) minus 63].^37^ Participants were considered to have diabetes if they had been previously diagnosed with diabetes by a health professional at the time of recruitment, if they were currently taking insulin or diabetes medication, or if they would be classified as having diabetes according to the World Health Organization guidelines for the glucose tolerance test (fasting venous plasma glucose ≥140 mg/dL or 2-hour venous plasma glucose ≥200 mg/dL).^38^ At follow-up, diabetes was diagnosed according to the same guidelines used at baseline.

### Statistical methods

We first examined univariate distributions of urinary W, outcomes, and covariates in the full analytic sample (n = 1,609; Figure 1). Based on these distributions (eFigures 1 and 2; http://links.lww.com/EE/A156), we natural log-transformed values for metal concentrations (μg/L), fasting glucose (FG), 2-hour glucose (FG-2hr), HOMA-IR, and HOMA-β. We then examined the Pearson correlations among baseline exposure levels of the metals (W, arsenic, cadmium, and lead). Next, we compared proportions (using chi-squared tests for categorical variables) and means (using two-sample t-tests for continuous variables) of each outcome and covariate by urinary W (below or above the median of 0.22 µg/L) at baseline. We also used chi-squared tests and two-sample t-tests with equal variances to assess whether participant characteristics were different among those missing either fasting glucose or fasting insulin (n = 178) and those missing neither (n = 1,431). Finally, we used these tests to assess the differences between baseline categorical and continuous covariate measurements, respectively, for participants who developed diabetes (n = 119) and those who did not (n = 959).

To assess the linearity assumption and evaluate potential effect modifiers, we first used Bayesian kernel machine regression (BKMR) to estimate the association among log-transformed metal values (i.e., W, antimony, arsenic, barium, cadmium, cesium, chromium, cobalt, copper, lead, manganese, molybdenum, plutonium, selenium, thallium, uranium, and zinc) and odds of diabetes at baseline. We excluded metals with greater than 50% of samples below the limit of detection (i.e., plutonium and uranium). We evaluated whether lnW was cross-sectionally associated with each outcome variable at the baseline examination cycle using linear regression (for lnFG, lnFG-2hr, lnHOMA-IR, and lnHOMA-β) and logistic regression (for diabetes prevalence). We evaluated whether lnW was longitudinally associated with each outcome variable among the participants without diabetes at baseline using linear mixed effect models with a random intercept for each participant (for lnFG, lnFG-2hr, lnHOMA-IR, and lnHOMA-β) and Fine and Gray competing risks regression (for incident diabetes, competing event = all-cause mortality). Although the proportional hazards assumption was not met for the Fine and Gray competing risks regression (*p* = 0.001), our sample size was large (n > 1,000) and the Kaplan-Meier survival estimates seemed to approximately follow the proportional hazards assumption beyond 10 years (eFigure 3; http://links.lww.com/EE/A156), so we assumed that the modeling method was still appropriate. To assess whether sex or ethnicity modified associations, we ran sex- and ethnicity-stratified models (separately). As a sensitivity analysis to reduce the potential for error in W estimation, we estimated the primary cross-sectional and longitudinal models excluding participants with baseline W concentrations below the limit of detection (n = 525 at baseline). Finally, we included a sensitivity analysis of longitudinal models using quartiles of urinary W rather than log-transformed values as the exposure.

All multivariable models were estimated twice—once each with two sets of covariates determined *a priori* using an evidence-based directed acyclic graph (DAG; eFigure 4; http://links.lww.com/EE/A156).^39^ The set of covariates in the main models and the BKMR model included baseline values of age (included as the unit of time for the mixed effect models), sex (excluded in the models stratified by sex), ethnicity (Hispanic, not Hispanic; excluded in models stratified by ethnicity), education (<12 years, 12 years, >12 years of schooling), smoking status (never, former, current), hypertension, body mass index (BMI; kg/m^2^), caloric intake (kcal/day), alcohol intake (g/week), and urinary creatinine levels (g/L). The set of covariates included in the further adjusted models also included natural log-transformed baseline values of arsenic (lnAs), cadmium (lnCd), and lead (lnPb).

## Results

Characteristics of the participants at baseline are described in Table 1. At baseline, the median W concentration was 0.22 μg/L (interquartile range [IQR] = 0.20, 0.59; see eTable 1; http://links.lww.com/EE/A156 for baseline distribution of all urinary metal exposures). In bivariate analyses, having urinary W above the median was significantly associated with being male, non-Hispanic, a current/former smoker, having differing levels of attained education, higher alcohol intake, lower fasting glucose, lower 2-hour glucose, and lower diabetes prevalence (Table 1). Baseline lnW concentrations were statistically significantly correlated with lnAs (r = 0.51) and weakly (but significantly) correlated with lnCd and lnPb levels (r = 0.16, 0.08, respectively; eTable 2; http://links.lww.com/EE/A156). Participant characteristics were generally similar among those with and without HOMA-IR or HOMA-β values, although we observed significant differences for ethnicity and BMI (eTable 3; http://links.lww.com/EE/A156).

**Table 1.** Baseline characteristics of the study sample stratified by tungsten (W) concentrations above and below the median

	Alln (%) or mean (standard deviation)	W ≤ 0.22 µg/Ln (%) or mean (standard deviation)	W > 0.22 µg/Ln (%) or mean (standard deviation)	*P* value for difference by urinary W group^a^

Total	1,609 (100)	805 (50.0)	804 (50.0)	
Age	54.3 (12.2)	54.7 (12.3)	53.8 (12.1)	0.120
Sex				<0.001*
Men	754 (46.9)	330 (41.0)	424 (52.7)	
Women	855 (53.1)	475 (59.0)	380 (47.3)	
Ethnicity				<0.001*
Hispanic	773 (48.0)	460 (57.1)	313 (38.9)	
Non-Hispanic	836 (52.0)	345 (42.9)	491 (61.1)	
Education				<0.001*
<12 years	526 (32.8)	302 (37.5)	224 (28.0)	
12 years	540 (33.6)	271 (33.7)	269 (33.6)	
>12 years	539 (33.6)	232 (28.8)	307 (38.4)	
Smoking status^b^				0.001*
Never	721 (44.9)	398 (49.4)	323 (40.3)	
Current	387 (24.1)	172 (21.4)	215 (26.8)	
Former	499 (31.1)	235 (29.2)	264 (32.9)	
Hypertension prevalence	627 (39.0)	322 (40.1)	305 (38.0)	0.396
Body mass index (kg/m^2^)	26.7 (4.81)	26.9 (4.99)	26.5 (4.62)	0.127
Caloric intake (kcal/day)^c^	1,510 (578)	1,523 (594)	1,498 (562)	0.388
Alcohol (g/week)^c^	41.0 (104)	31.5 (91.7)	50.6 (115)	<0.001*
Fasting glucose (mg/dL)^d^	120 (56.0)	122 (61.0)	117 (50.3)	0.039*
2-hour glucose (mg/dL)	154 (101)	160 (106)	148 (94.7)	0.026*
Fasting insulin (μU/mL)	14.6 (10.5)	14.4 (9.83)	14.8 (11.0)	0.434
HOMA-IR^e^	4.19 (4.13)	4.20 (4.21)	4.18 (4.05)	0.938
HOMA-β^e^	7.65 (8.41)	7.69 (10.3)	7.61 (6.15)	0.858
Diabetes prevalence^f^	457 (28.4)	249 (30.9)	208 (25.9)	0.024*
Study time to death, diabetes, or censoring (years)^g^	10.4 (5.14)	10.3 (4.92)	10.4 (5.35)	0.791

*Significant with p < 0.05

^a^Differences between baseline categorical and continuous covariate measurements were assessed using chi-squared and t-tests, respectively, among those below or at the median W value and above the median W value.

^b^Smoking status of never was defined as <100 cigarettes in lifetime. Smoking status of current was defined as ≥100 cigarettes in lifetime and currently a smoker. Smoking status of ever was defined as ≥100 cigarettes in lifetime and not currently a smoker.

^c^Caloric intake and alcohol intake were measured using a food frequency questionnaire.

^d^SI conversion factors: to convert mg/dL to mmol/L, multiply by 0.056. To convert μU/mL to pmol/L, multiply by 0.144.

^e^Homeostatic Model Assessment of Insulin Resistance (HOMA-IR) was calculated as the product of fasting insulin (μU/mL) and fasting glucose (mg/dL) divided by 405. Homeostatic Model Assessment of beta cell function (HOMA-β) was calculated as [20 times fasting insulin (μU/mL)] / [fasting glucose (mg/dL) minus 63].

^f^People with diabetes were currently taking insulin or oral hypoglycemic, were diagnosed according to the oral glucose tolerance test,^38^ or had previously been diagnosed with diabetes.

^g^Calculated only among participants who did not have diabetes at baseline (n = 1,078).

Of the 1,078 participants without diabetes at baseline, 119 developed diabetes during the study period (mean time at risk = 9.9 years) and 301 participants died before developing diabetes (mean time to death = 14.0 years). The mean follow-up time before a censored event was 10.4 years. Compared with participants who did not develop diabetes, those who developed diabetes were significantly more likely to be older, Hispanic, hypertensive, have differing levels of attained education, and have a higher BMI (eTable 4; http://links.lww.com/EE/A156).

The assumption of linearity seemed reasonable as lnW appeared to be linearly associated with odds of diabetes at baseline except at the highest lnW concentrations (eFigure 5; http://links.lww.com/EE/A156). Tables 2 and 3 show the effect estimates cross-sectionally relating lnW to lnFG, lnFG-2hr, lnHOMA-IR, lnHOMA-β, and diabetes prevalence at baseline. Although lnW was not significantly associated with lnFG, lnFG-2hr, lnHOMA-β, or diabetes prevalence, a doubling of urinary W was significantly associated with 3% higher lnHOMA-IR in the main model overall (95% confidence interval [CI] = 1 to 5 higher), 5% higher among males (95% CI = 2 to 9 higher), and 5% higher among Hispanics (95% CI = 1 to 9 higher; Table 2 and eTable 5; http://links.lww.com/EE/A156). The associations with lnHOMA-IR were not significant in models adjusting for other metals (Table 2 and eTable 5; http://links.lww.com/EE/A156). In a sensitivity analysis excluding participants with urinary W values below the limit of detection and adjusting for exposure to other metals, lnW was significantly associated with 2% decreased lnFG (95% CI = <1 to 3 lower) and 16% lower diabetes prevalence (odds ratio = 0.84; 95% CI = 0.71 to 0.99; eTable 6; http://links.lww.com/EE/A156). In a multipollutant BKMR analysis, antimony, arsenic, and copper appeared to be effect modifiers of the association between lnW and odds of diabetes (eFigure 5; http://links.lww.com/EE/A156).

**Table 2.** Associations between urinary tungsten and continuous diabetes measures

	Cross-sectional associationsβ (95% CI)	Longitudinal associationsβ (95% CI)
**Natural-log transformed fasting glucose**	*n = 1,567*	*n = 1,055*
Main model^a^	0.002 (-0.013, 0.017)	0.008 (0.002, 0.014)*
Further adjusted model^b^	-0.013 (-0.030, 0.004)	0.007 (0.000, 0.013)*
**Natural-log transformed 2-hour glucose**	*n = 1,485*	*n = 1,050*
Main model	0.005 (-0.018, 0.029)	0.011 (-0.005, 0.028)
Further adjusted model	-0.012 (-0.038, 0.014)	0.018 (-0.002, 0.037)
**Natural-log transformed HOMA-IR^c^**	*n = 1,401*	*n = 1,054*
Main model	0.043 (0.011, 0.076)*	0.045 (0.022, 0.069)*
Further adjusted model	0.027 (-0.010, 0.063)	0.041 (0.014, 0.068)*
**Natural-log transformed HOMA-β^c^**	*n = 1,399*	*n = 1,054*
Main model	0.018 (-0.014, 0.050)	0.018 (-0.007, 0.042)
Further adjusted model	0.028 (-0.008, 0.064)	0.020 (-0.009, 0.048)

*Significant with *p* < 0.05.

^a^Main model adjusted for age (years), sex, ethnicity (Hispanic/non-Hispanic), education (<12/12/>12 years), smoking status (current/former/never), hypertension (dichotomous), body mass index (kg/m^2^), caloric intake (kcal/day), alcohol intake (g/week), and urinary creatinine (g/L).

^b^Further adjusted model adjusted for all covariates in the main model and also natural log-transformed arsenic, cadmium, and lead.

^c^Homeostatic Model Assessment of Insulin Resistance (HOMA-IR) = [fasting insulin (μU/mL) × fasting glucose (mg/dL)]/405; Homeostatic Model Assessment of beta cell function (HOMA-β) = [20 × fasting insulin (μU/mL)]/[fasting glucose (mg/dL) – 63].^37^

**Table 3.** Associations between urinary tungsten and diabetes

	Cross-sectional associations with diabetes prevalenceOR (95% CI)n = 1,573	Longitudinal associations with diabetes incidenceSHR (95% CI)n = 1,056
Main model^a^	0.98 (0.87, 1.12)	1.28 (1.09, 1.50)*
Further adjusted model^b^	0.87 (0.75, 1.01)	1.24 (1.03, 1.48)*

*Significant with *p* < 0.05.

^a^Main model adjusted for age (years; treated as time variable in Fine and Gray competing risks regression models), sex, ethnicity (Hispanic/non-Hispanic), education (<12/12/>12 years), smoking status (current/former/never), hypertension (dichotomous), body mass index (kg/m^2^), caloric intake (kcal/day), alcohol intake (g/week), and urinary creatinine (g/L).

^b^Further adjusted model adjusted for all covariates in the Main model and also natural log-transformed arsenic, cadmium, and lead.

CI indicates confidence intervals; OR, odds ratio; SHR, subdistribution hazard ratio.

In longitudinal analyses, lnW was significantly associated with a 1% increased lnFG (main model: 95% CI = <1 to 1 increased; further adjusted model: <1% increased, 95% CI = 0 to 1 increase), 3% increased lnHOMA-IR (main model: 95% CI = 2 to 5 increase; further adjusted model: 3% increase, 95% CI = 1 to 5 increase), and 28% higher incident diabetes (main model subdistribution hazard ratio [SHR] = 1.28; 95% CI = 1.09 to 1.50; further adjusted model SHR = 1.24; 95% CI = 1.03 to 1.48; Tables 2 and 3). The strength of these associations was similar in sensitivity analyses excluding participants with W values below the limit of detection, although the association with lnFG became nonsignificant (eTable 6; http://links.lww.com/EE/A156). Sex-stratified models indicated some effect measure modification, but several of the associations became nonsignificant (though with similar effect estimates to the nonstratified models; Table 4 and eTable 7; http://links.lww.com/EE/A156). In ethnicity-stratified models for lnFG, lnFG-2hr, and lnHOMA-IR, we observed slightly stronger associations among Hispanics than non-Hispanics (Table 4). Conversely, in the ethnicity-stratified models for incident diabetes, we observed somewhat stronger associations among non-Hispanics (eTable 7; http://links.lww.com/EE/A156). When examining the association using urinary W categorized into quartiles, the longitudinal associations remained significant (diabetes *P* value for trend = 0.004; lnFG *P* value for trend = 0.016; lnHOMA-IR *P* value for trend = 0.003).

**Table 4.** Longitudinal associations between tungsten and continuous diabetes measures stratified by sex and ethnicity^a^

	Malesβ (95% CI)	Femalesβ (95% CI)	Non-Hispanicsβ (95% CI)	**Hispanics**β (95% CI)
**Natural-log transformed fasting glucose**	n = 501	n = 554	n = 620	n = 435
Main model^b^	0.007 (–0.001, 0.015)	0.009 (0.000, 0.017)*	0.006 (–0.001, 0.013)	0.014 (0.002, 0.025)*
Further adjusted model^c^	0.005 (–0.004, 0.014)	0.008 (–0.001, 0.017)	0.003 (-0.005, 0.010)	0.016 (0.003, 0.030)*
**Natural-log transformed 2-hour glucose**	n = 500	n = 550	n = 618	n = 432
Main model	0.022 (–0.004, 0.047)	0.001 (–0.021, 0.023)	0.008 (–0.012, 0.028)	0.023 (–0.009, 0.054)
Further adjusted model	0.018 (–0.012, 0.047)	0.015 (–0.011, 0.040)	0.010 (–0.012, 0.032)	0.038 (0.001, 0.076)*
**Natural-log transformed HOMA-IR** ^d^	n = 501	n = 553	n = 620	n = 434
Main model	0.050 (0.019, 0.082)*	0.036 (0.001, 0.071)*	0.033 (0.005, 0.061)*	0.076 (0.035, 0.118)*
Further adjusted model	0.036 (–0.001, 0.074)	0.038 (–0.001, 0.076)	0.025 (–0.009, 0.058)	0.083 (0.037, 0.130)*
**Natural-log transformed HOMA-β** ^d^	n = 501	n = 553	n = 620	n = 434
Main model	0.026 (–0.009, 0.061)	0.005 (–0.030, 0.041)	0.016 (–0.012, 0.043)	0.021 (–0.028, 0.070)
Further adjusted model	0.019 (–0.022, 0.060)	0.013 (–0.026, 0.052)	0.020 (–0.012, 0.053)	0.021 (–0.033, 0.075)

*Significant with *p* < 0.05.

^a^Stratified models did not include the variable stratified on as a covariate.

^b^Main model adjusted for age (years; treated as time variable in Fine and Gray competing risks regression models), sex, ethnicity (Hispanic/non-Hispanic), education (<12/12/>12 years), smoking status (current/former/never), hypertension (dichotomous), body mass index (kg/m^2^), caloric intake (kcal/day), alcohol intake (g/week), and urinary creatinine (g/L).

^c^Further adjusted model adjusted for all covariates in the Main model and also natural log-transformed arsenic, cadmium, and lead.

^d^Homeostatic Model Assessment of Insulin Resistance (HOMA-IR) = [fasting insulin (μU/mL) × fasting glucose (mg/dL)]/405; Homeostatic Model Assessment of beta cell function (HOMA-β) = [20 × fasting insulin (μU/mL)]/[fasting glucose (mg/dL) – 63].

## Discussion

We presented a longitudinal analysis investigating the associations between urinary W, fasting glucose, 2-hour glucose, measures of insulin resistance and β-cell function, and incident diabetes. Our analysis is the first to examine these associations accounting for coexposure to other metals and the first to examine sex- and ethnicity-stratified associations. We observed that a doubling of urinary lnW was cross-sectionally associated with 3% higher lnHOMA-IR and longitudinally associated with 1% higher lnFG and 3% higher lnHOMA-IR. Similarly, each natural log-unit increase in urinary W was associated with a 28% increased risk of developing diabetes over the follow-up period. These associations were robust when adjusting for the set of covariates suggested by an evidence-based DAG,^39^ and when further adjusting for coexposure to arsenic, cadmium, and lead. Although future longitudinal studies are needed to validate our results in other populations, our findings are timely given the diabetes epidemic in the United States,^1^ current efforts to develop an oral antidiabetic treatment option that contains tungstate,^10,40,41^ and ongoing low-level occupational exposure to W.^42,43^

Despite differences in study designs, populations, and exposure levels, our primary findings were in agreement with previous cross-sectional studies.^6,20^ Given the differences in study population, the results might be transportable to other populations (our study population was drawn from individuals in one rural location compared with the previous studies that included a sample of adults in one urban region of China^6^ and in a nationally representative sample of US adults).^20^ Similarly, given the differences in exposure levels, we might expect the associations we observed to extrapolate to somewhat lower exposure levels (our participants were exposed to median urinary W values of 0.22 μg/L compared with median values of 0.12 μg/L and 0.07 µg/L in the two cross-sectional studies).^6,20^ Finally, the associations may not be time period-specific: our study included data collected beginning in 1984 whereas the others collected data beginning in 2011 and 1999, respectively.^6,20^ This difference in time period could also partially explain the difference in urinary W levels, though notably the SLVDS urinary W levels were lower than the geometric mean in a nationally representative sample of US adults for that time period (95% CI = 0.31 to 0.34 µg/L in our population versus 95% CI = 0.63 to 0.77 µg/L nationally from 1988 to 1994).^44^ Another prospective study conducted in US women did not find a significant association between urinary W and incident diabetes; however, this study used above or below the limit of detection as exposed or unexposed, respectively, and only 29% of participants had detectable W levels.^21^ Thus, the differences in exposure assessment could explain the discrepancies in results.

Relatively sparse literature exists that would suggest potential mechanisms through which W may affect the development of diabetes. The results from our study indicate that W could influence diabetes risk through insulin resistance rather than β-cell function—though insulin resistance and β-cell dysfunction often interact in the development of diabetes,^45^ and it is possible that the measurement methods for HOMA-β were less accurate (especially for participants on insulin medication).^46^ More generally, W can be pro-inflammatory,^47^ and inflammation can lead to endothelial dysfunction and insulin resistance.^48^ This could partially explain the association between W and diabetes incidence as well as between W and HOMA-IR. The association between W and hyperglycemia could also be caused by endothelial dysfunction, as W can affect this process through inhibition of a related antioxidant molybdoenzyme.^49–52^ Additionally, it is possible that other trace metals interact with W (or similarly to W) in its association with diabetes. For example, chromium, another group six transition metal with a high melting point, is involved with the metabolism of glucose and insulin.^53,54^

Furthermore, there could be sex-differences underlying the mechanisms relating urinary W to diabetes. For example, one study observed that the effect of W on bone homeostasis in mice was sex-dependent.^55^ Similarly, sex-related hormonal differences seemed to affect the absorption rate of other metals in humans.^56^ Additionally, the mechanisms through which individuals develop diabetes vary by sex and this could partially explain sex differences in our results. For example, consistent with our observation that associations with HOMA-IR were stronger in men than women, males are more likely to have insulin resistance, whereas females are more likely to have impaired glucose tolerance.^23,24^

Our study had several strengths. These strengths include the longitudinal nature of the analysis, the inclusion of multiple diabetes markers indicative of different possible roles of W in the pathophysiology of diabetes, the ability to adjust for covariates determined through an evidence-based DAG, the ability to adjust for coexposure to toxic metals, and the assessment of sex- and ethnicity-related effect modification. Other strengths include the use of urinary metal measurements (a common method to estimate chronic exposure to most heavy metals),^57^ large sample size, low attrition rate, and exposure contrast in our sample. Furthermore, any measurement error in urinary W would likely be nondifferential (biasing the results toward the null) since measurement error would not depend on diabetes status.

Our study also had several limitations. For example, we used the oral glucose tolerance test rather than robust measures of whole body insulin resistance and β-cell function (e.g., the hyperglycemic or hyperinsulinemic-euglycemic clamp, or the frequently sampled intravenous glucose tolerance test) to assess HOMA-IR, HOMA-β, and plasma glucose levels.^46,58,59^ Nonetheless, we observed significant associations with other markers of diabetes in addition to insulin resistance calculated through the HOMA-IR model. We also used the WHO criteria from 1985 to classify participants with diabetes, as this was the criteria used at the time of data collection. This definition required a fasting glucose level of at least 140 mg/dL to diagnose diabetes,^38^ whereas current guidelines recommend a cutoff of 126 mg/dL.^60^ We used the guidelines at the time of data collection to follow the clinical decision-making process at the time. Nonetheless, we recognize that there could have been undiagnosed cases of diabetes. It is possible that our outcome misclassification was differential, in part due to missing data patterns whereby participants who identified as non-Hispanic and who had a higher BMI were more likely to be missing fasting glucose and fasting insulin outcome data. No other characteristics significantly varied between individuals with and without outcome data.

Additionally, we used urinary W levels measured at baseline, and we did not have data on time-varying changes in exposure. This may be a limitation since the majority (approximately 60% of daily intake) of ingested W is rapidly excreted through urine within a day.^11^ However, urinary W levels were highly correlated with W concentrations in drinking water for this study population (results not shown), and human intake of drinking water, although daily amounts may vary, is likely to remain relatively stable over longer periods due to the biologic requirement of water.^61^ Thus, we postulate that W levels excreted through urine may have also been relatively stable over time. Other biomarkers of arsenic and lead, such as hair and nail, are stronger biomarkers of long-term exposure.^62,63^ On the other hand, urinary cadmium is a strong biomarker of long-term cadmium exposure,^64^ and urinary measures of these other metals have been found suitable for biomonitoring.^62,63^

Other limitations include our assumption that the longitudinal exposure-response function was linear (as the BKMR analysis suggested that the cross-sectional exposure-response function was). Longitudinal trends were similar when using quartiles of W as the exposure, which reduces concern of nonlinearity; however, as suggested by the BKMR results, there is still potential for a nonlinear association at higher lnW concentrations. We also did not account for potential confounding by certain other elements (e.g., nickel). We could not assess confounding by nickel, as this metal was not included in the metals panel. Since W has been suggested to increase negative health effects of nickel,^10^ there is still potential for unmeasured confounding. Finally, urinary W has been previously shown to be associated with chronic kidney disease in this population.^19^ Because we measured W in urine, it is possible that impaired kidney function could alter the urinary excretion of W,^65^ leading to misclassification of W measurements among those with kidney damage. However, we decided to not account for kidney function given the observed cyclical relationship between diabetes and kidney function.^66,67^

Although future studies are needed to examine how time-varying urinary W levels affect diabetes incidence in this population and in other populations, our longitudinal study suggests that exposure to W is associated with increased fasting glucose levels, HOMA-IR levels, and diabetes incidence. Given that W has industrial applications^10^ and exposure disparities persist,^68^ our results could inform future diabetes prevention efforts.

## Conflicts of interest statement

The authors declare that they have no conflicts of interest with regard to the content of this report.

## Acknowledgments

We would like to thank the participants and staff of the San Luis Valley Diabetes Study. All the intellectual property and data generated were administered according to policies from the University of Colorado and the NIH, including the NIH Data Sharing Policy and Implementation Guidance of March 5, 2003. The San Luis Valley Diabetes Study (SLVDS) summary data can be shared with the scientific community in accordance with the SLVDS data sharing plan (which includes Data Use Agreements and anonymizing of data to protect subject confidentiality). Analytic code is available upon reasonable request from the corresponding author.

## Supplementary Material



## References

[1] Centers for Disease Control and Prevention. National Diabetes Statistics Report 2020. US Dep Health Hum Serv. 2020:32.

[2] Krishna S Gillespie KN McBride TM. Diabetes burden and access to preventive care in the rural United States. J Rural Health. 2010;26:3–11. 20105262 10.1111/j.1748-0361.2009.00259.x

[3] O’Connor A Wellenius G. Rural-urban disparities in the prevalence of diabetes and coronary heart disease. Public Health. 2012;126:813–820. 22922043 10.1016/j.puhe.2012.05.029

[4] Murea M Ma L Freedman BI. Genetic and environmental factors associated with type 2 diabetes and diabetic vascular complications. Rev Diabet Stud. 2012;9:6–22. 22972441 10.1900/RDS.2012.9.6 PMC3448170

[5] Wang W Xie Z Lin Y Zhang D. Association of inorganic arsenic exposure with type 2 diabetes mellitus: a meta-analysis. J Epidemiol Community Health. 2014;68:176–184. 24133074 10.1136/jech-2013-203114

[6] Feng W Cui X Liu B. Association of urinary metal profiles with altered glucose levels and diabetes risk: a population-based study in China. PLoS ONE. 2015;10:e0123742. 25874871 10.1371/journal.pone.0123742 PMC4395404

[7] Khan F Momtaz S Niaz K Hassan FI Abdollahi M. Epigenetic mechanisms underlying the toxic effects associated with arsenic exposure and the development of diabetes. Food Chem Toxicol. 2017;107(Pt A):406–417. 28709971 10.1016/j.fct.2017.07.021

[8] Edwards J Ackerman C. A review of diabetes mellitus and exposure to the environmental toxicant cadmium with an emphasis on likely mechanisms of action. Curr Diabetes Rev. 2016;12:252–258. 26264451 10.2174/1573399811666150812142922 PMC5002940

[9] Khan AR Awan FR. Metals in the pathogenesis of type 2 diabetes. J Diabetes Metab Disord. 2014;13:16. 24401367 10.1186/2251-6581-13-16 PMC3916582

[10] Bolt AM Mann KK. Tungsten: an Emerging Toxicant, Alone or in Combination. Curr Environ Health Rep. 2016;3:405–415. 27678292 10.1007/s40572-016-0106-z

[11] Agency for Toxic Substances and Disease Registry. Toxicological Profile for Tungsten. US Public Health Serv. 2005:203. 38147521

[12] Shah Idil A Donaldson N. The use of tungsten as a chronically implanted material. J Neural Eng. 2018;15:021006. 29300000 10.1088/1741-2552/aaa502

[13] Keith LS Wohlers DW Moffett DB Rosemond ZA; Agency for Toxic Substances and Disease Registry. ATSDR evaluation of potential for human exposure to tungsten. Toxicol Ind Health. 2007;23:309–345. 18386524 10.1177/0748233707081906

[14] Drinking Water Regulations. United States Environmental Protection Agency. 2015. Available at: https://www.epa.gov/dwreginfo/drinking-water-regulations. Accessed September 22, 2020.

[15] US EPA. Emerging Contaminants and Federal Facility Contaminants of Concern. 2013. Available at: https://www.epa.gov/fedfac/emerging-contaminants-and-federal-facility-contaminants-concern. Accessed November 19, 2019.

[16] Nominated Substances. National Toxicology Program. 2019. Available at: https://ntp.niehs.nih.gov/getinvolved/nominate/substances/index.html. Accessed November 19, 2019.

[17] Shiue I Hristova K. Higher urinary heavy metal, phthalate and arsenic concentrations accounted for 3-19% of the population attributable risk for high blood pressure: US NHANES, 2009-2012. Hypertens Res. 2014;37:1075–1081. 25077919 10.1038/hr.2014.121

[18] Tyrrell J Galloway TS Abo-Zaid G Melzer D Depledge MH Osborne NJ. High urinary tungsten concentration is associated with stroke in the National Health and Nutrition Examination Survey 1999–2010. PLoS ONE. 2013;8:e77546. 24244278 10.1371/journal.pone.0077546 PMC3823878

[19] Fox J Macaluso F Moore C. Urine tungsten and chronic kidney disease in rural Colorado. Environ Res. 2021;195:110710. 33460634 10.1016/j.envres.2021.110710 PMC7987874

[20] Menke A Guallar E Cowie CC. Metals in urine and diabetes in U.S. adults. Diabetes. 2016;65:164–171. 26542316 10.2337/db15-0316 PMC4686948

[21] Wang X Karvonen-Gutierrez CA Herman WH Mukherjee B Harlow SD Park SK. Urinary metals and incident diabetes in midlife women: study of Women’s Health Across the Nation (SWAN). BMJ Open Diabetes Res Care. 2020;8:e001233. 10.1136/bmjdrc-2020-001233 PMC7398092 32747380

[22] Kautzky-Willer A Harreiter J Pacini G. Sex and gender differences in risk, pathophysiology and complications of type 2 diabetes mellitus. Endocr Rev. 2016;37:278–316. 27159875 10.1210/er.2015-1137 PMC4890267

[23] Arnetz L Ekberg NR Alvarsson M. Sex differences in type 2 diabetes: focus on disease course and outcomes. Diabetes Metab Syndr Obes. 2014;7:409–420. 25258546 10.2147/DMSO.S51301 PMC4172102

[24] Gannon M Kulkarni RN Tse HM Mauvais-Jarvis F. Sex differences underlying pancreatic islet biology and its dysfunction. Mol Metab. 2018;15:82–91. 29891438 10.1016/j.molmet.2018.05.017 PMC6066785

[25] Velasco-Mondragon E Jimenez A Palladino-Davis AG Davis D Escamilla-Cejudo JA. Hispanic health in the USA: a scoping review of the literature. Public Health Rev. 2016;37:31. 29450072 10.1186/s40985-016-0043-2 PMC5809877

[26] James KA Meliker JR Marshall JA Hokanson JE Zerbe GO Byers TE. Validation of estimates of past exposure to arsenic in drinking water using historical urinary arsenic concentrations. J Expo Sci Environ Epidemiol. 2013;23:450–454. 23443236 10.1038/jes.2013.8

[27] James KA Byers T Hokanson JE Meliker JR Zerbe GO Marshall JA. Association between lifetime exposure to inorganic arsenic in drinking water and coronary heart disease in Colorado residents. Environ Health Perspect. 2015;123:128–134. 25350952 10.1289/ehp.1307839 PMC4314243

[28] San Luis Valley Development Resources Group & Council of Governments. 2021 San Luis Valley Statistical Profile. 2021. SLVDRG San Luis Valley Development Resource Group Alamosa Colorado. Available at: https://www.slvdrg.org/research-data/. Accessed April 9, 2021.

[29] Hamman RF Marshall JA Baxter J. Methods and prevalence of non-insulin-dependent diabetes mellitus in a biethnic Colorado population. The San Luis Valley Diabetes Study. Am J Epidemiol. 1989;129:295–311. 2912042 10.1093/oxfordjournals.aje.a115134

[30] Unger T Borghi C Charchar F. 2020 International Society of Hypertension global hypertension practice guidelines. Hypertension. 2020;75:1334–1357. 32370572 10.1161/HYPERTENSIONAHA.120.15026

[31] Marshall JA Weiss NS Hamman RF. The role of dietary fiber in the etiology of non-insulin-dependent diabetes mellitus. The San Luis Valley Diabetes Study. Ann Epidemiol. 1993;3:18–26. 8287151 10.1016/1047-2797(93)90005-o

[32] Rivera-Núñez Z Meliker JR Meeker JD Slotnick MJ Nriagu JO. Urinary arsenic species, toenail arsenic, and arsenic intake estimates in a Michigan population with low levels of arsenic in drinking water. J Expo Sci Environ Epidemiol. 2012;22:182–190. 21878987 10.1038/jes.2011.27 PMC10037220

[33] Delanghe JR Speeckaert MM. Creatinine determination according to Jaffe-what does it stand for? NDT Plus. 2011;4:83–86. 25984118 10.1093/ndtplus/sfq211 PMC4421578

[34] Beckman Instruments. Glucose Analyzer 2 Operating Manual. OSR General Chemistry; 1988.

[35] Desbuquois B Aurbach GD. Use of polyethylene glycol to separate free and antibody-bound peptide hormones in radioimmunoassays. J Clin Endocrinol Metab. 1971;33:732–738. 5166455 10.1210/jcem-33-5-732

[36] Marshall JA Bessesen DH Hamman RF. High saturated fat and low starch and fibre are associated with hyperinsulinaemia in a non-diabetic population: the San Luis Valley Diabetes Study. Diabetologia. 1997;40:430–438. 9112020 10.1007/s001250050697

[37] Matthews DR Hosker JP Rudenski AS Naylor BA Treacher DF Turner RC. Homeostasis model assessment: insulin resistance and beta-cell function from fasting plasma glucose and insulin concentrations in man. Diabetologia. 1985;28:412–419. 3899825 10.1007/BF00280883

[38] WHO Study Group on Diabetes Mellitus, Organization WH. Diabetes mellitus: report of a WHO study group [meeting held in Geneva from 11 to 16 February 1985]. World Health Organization; 1985. Available at: https://apps.who.int/iris/handle/10665/39592. Accessed September 18, 2020.

[39] Riseberg E Melamed RD James KA Alderete TL Corlin L. Development and application of an evidence-based directed acyclic graph to evaluate the associations between metal mixtures and cardiometabolic outcomes [published online ahead of print March 8, 2021]. medRxiv. 2021.03.05.21252993. doi:10.1101/2021.03.05.21252993 10.1515/em-2022-0133 PMC10292771 37377511

[40] Altirriba J Barbera A Del Zotto H. Molecular mechanisms of tungstate-induced pancreatic plasticity: a transcriptomics approach. BMC Genomics. 2009;10:406. 19715561 10.1186/1471-2164-10-406 PMC2741493

[41] Bertinat R Westermeier F Gatica R Nualart F. Sodium tungstate: is it a safe option for a chronic disease setting, such as diabetes? J Cell Physiol. 2018;234:51–60. 30132852 10.1002/jcp.26913

[42] Paganelli M Fostinelli J Renzetti S. Occupational low-level exposure to hard metals: cobalt and tungsten biomonitoring as an effective tool to evaluate the effectiveness of industrial hygiene interventions for risk management. Biomarkers. 2020;25:179–185. 31996048 10.1080/1354750X.2020.1724195

[43] Klasson M Bryngelsson IL Pettersson C Husby B Arvidsson H Westberg H. Occupational exposure to cobalt and tungsten in the swedish hard metal industry: air concentrations of particle mass, number, and surface area. Ann Occup Hyg. 2016;60:684–699. 27143598 10.1093/annhyg/mew023 PMC4915521

[44] Paschal DC Ting BG Morrow JC. Trace metals in urine of United States residents: reference range concentrations. Environ Res. 1998;76:53–59. 9466897 10.1006/enrs.1997.3793

[45] Cerf ME. Beta cell dysfunction and insulin resistance. Front Endocrinol (Lausanne). 2013;4:37. 23542897 10.3389/fendo.2013.00037 PMC3608918

[46] Wallace TM Levy JC Matthews DR. Use and abuse of HOMA modeling. Diabetes Care. 2004;27:1487–1495. 15161807 10.2337/diacare.27.6.1487

[47] Byrne JV Hope JKA Hubbard N Morris JH. The nature of thrombosis induced by platinum and tungsten coils in saccular aneurysms. AJNR Am J Neuroradiol. 1997;18:29–33. 9010517 PMC8337884

[48] Hartge MM Unger T Kintscher U. The endothelium and vascular inflammation in diabetes. Diab Vasc Dis Res. 2007;4:84–88. 17654441 10.3132/dvdr.2007.025

[49] Navas-Acien A Silbergeld EK Sharrett R Calderon-Aranda E Selvin E Guallar E. Metals in urine and peripheral arterial disease. Environ Health Perspect. 2005;113:164–169. 15687053 10.1289/ehp.7329 PMC1277859

[50] Rask-Madsen C King GL. Mechanisms of Disease: endothelial dysfunction in insulin resistance and diabetes. Nat Clin Pract Endocrinol Metab. 2007;3:46–56. 17179929 10.1038/ncpendmet0366

[51] Berry CE Hare JM. Xanthine oxidoreductase and cardiovascular disease: molecular mechanisms and pathophysiological implications. J Physiol. 2004;555(pt 3):589–606. 14694147 10.1113/jphysiol.2003.055913 PMC1664875

[52] Johnson JL Waud WR Cohen HJ Rajagopalan KV. Molecular basis of the biological function of molybdenum. Molybdenum-free xanthine oxidase from livers of tungsten-treated rats. J Biol Chem. 1974;249:5056–5061. 4368927

[53] Anderson RA. Chromium in the prevention and control of diabetes. Diabetes Metab. 2000;26:22–27. 10705100

[54] Agency for Toxic Substances and Disease Registry. Toxicological Profile for Chromium. Agency for Toxic Substances and Disease Registry; 2012:592. 24049864

[55] Bolt AM Grant MP Wu TH. Tungsten promotes sex-specific adipogenesis in the bone by altering differentiation of bone marrow-resident mesenchymal stromal cells. Toxicol Sci. 2016;150:333–346. 26865663 10.1093/toxsci/kfw008 PMC4900133

[56] Lee BK Kim Y. Sex-specific profiles of blood metal levels associated with metal-iron interactions. Saf Health Work. 2014;5:113–117. 25379323 10.1016/j.shaw.2014.06.005 PMC4213922

[57] Kakkar P Jaffery FN. Biological markers for metal toxicity. Environ Toxicol Pharmacol. 2005;19:335–349. 21783494 10.1016/j.etap.2004.09.003

[58] Coates PA Luzio SD Brunel P Owens DR. Comparison of estimates of insulin sensitivity from minimal model analysis of the insulin-modified frequently sampled intravenous glucose tolerance test and the isoglycemic hyperinsulinemic clamp in subjects with NIDDM. Diabetes. 1995;44:631–635. 7789626 10.2337/diab.44.6.631

[59] Cersosimo E Solis-Herrera C Trautmann ME Malloy J Triplitt CL. Assessment of pancreatic β-cell function: review of methods and clinical applications. Curr Diabetes Rev. 2014;10:2–42. 24524730 10.2174/1573399810666140214093600 PMC3982570

[60] Genuth SM Palmer JP Nathan DM. Classification and diagnosis of diabetes. In: Cowie CC Casagrande SS Menke A, eds. Diabetes in America. 3rd ed. National Institute of Diabetes and Digestive and Kidney Diseases (US); 2018. Available at: http://www.ncbi.nlm.nih.gov/books/NBK568014/. Accessed July 23, 2021. 33651569

[61] Armstrong LE Johnson EC. Water intake, water balance, and the elusive daily water requirement. Nutrients. 2018;10:E1928. 30563134 10.3390/nu10121928 PMC6315424

[62] Sanders T Liu Y Buchner V Tchounwou PB. Neurotoxic effects and biomarkers of lead exposure: a review. Rev Environ Health. 2009;24:15–45. 19476290 10.1515/reveh.2009.24.1.15 PMC2858639

[63] Marchiset-Ferlay N Savanovitch C Sauvant-Rochat MP. What is the best biomarker to assess arsenic exposure via drinking water? Environ Int. 2012;39:150–171. 22208756 10.1016/j.envint.2011.07.015

[64] Vacchi-Suzzi C Kruse D Harrington J Levine K Meliker JR. Is urinary cadmium a biomarker of long-term exposure in humans? A review. Curr Environ Health Rep. 2016;3:450–458. 27696280 10.1007/s40572-016-0107-y PMC5453507

[65] Satarug S Vesey DA Gobe GC. Kidney cadmium toxicity, diabetes and high blood pressure: the perfect storm. Tohoku J Exp Med. 2017;241:65–87. 28132967 10.1620/tjem.241.65

[66] Alicic RZ Rooney MT Tuttle KR. Diabetic kidney disease: challenges, progress, and possibilities. Clin J Am Soc Nephrol. 2017;12:2032–2045. 28522654 10.2215/CJN.11491116 PMC5718284

[67] Bockenhauer D Bichet DG. Pathophysiology, diagnosis and management of nephrogenic diabetes insipidus. Nat Rev Nephrol. 2015;11:576–588. 26077742 10.1038/nrneph.2015.89

[68] Centers for Disease Control and Prevention. Fourth National Report on Human Exposure to Environmental Chemicals Update. Department of Health and Human Services; 2019:866.

