# Supplementary material for: Multipollutant, longitudinal analysis of the association between urinary tungsten and incident diabetes in a rural population
Source: Environ Epidemiol. 2021 Oct 13;5(6):e173. doi: 10.1097/EE9.0000000000000173 (PMC8663879; doi:10.1097/EE9.0000000000000173)
Supplement: Supplementary file 1 [file ee9-5-e173-s001.docx]

**Supplemental Table 1. Distribution of urinary metal concentrations at baseline (n = 1609)**

|  | Minimum | 25^th^ percentile | Median | 75^th^ percentile | Maximum | Geometric mean | 95% confidence interval for the geometric mean | n (%) below the limit of detection |
| --- | --- | --- | --- | --- | --- | --- | --- | --- |
| Tungsten (µg/L) | 0.03 | 0.20 | 0.22 | 0.59 | 20.4 | 0.33 | 0.31, 0.34 | 525 (32.6) |
| Arsenic (µg/L) | 0.93 | 7.06 | 15.8 | 32.8 | 1435 | 16.3 | 15.5, 17.1 | 118 (7.33) |
| Barium (µg/L) | 0.17 | 1.23 | 2.17 | 4.26 | 1090 | 2.41 | 2.28, 2.55 | 23 (1.43) |
| Cadmium (µg/L) | 0.04 | 0.38 | 0.67 | 1.22 | 27.9 | 0.69 | 0.66, 0.71 | 76 (4.72) |
| Cobalt (µg/L) | 0.04 | 0.33 | 0.55 | 0.92 | 11.7 | 0.61 | 0.58, 0.63 | 30 (1.86) |
| Chromium (µg/L) | 0.04 | 0.40 | 0.44 | 1.56 | 13.4 | 0.75 | 0.71, 0.79 | 486 (30.2) |
| Cesium (µg/L) | 0.10 | 2.08 | 3.33 | 5.13 | 97.6 | 3.15 | 3.04, 3.26 | 1 (0.06) |
| Copper (µg/L) | 1.85 | 13.3 | 22.9 | 46.1 | 2820 | 25.1 | 24.0, 26.4 | 14 (0.87) |
| Lead (µg/L) | 0.10 | 1.40 | 2.73 | 7.64 | 150 | 3.37 | 3.18, 3.58 | 24 (1.49) |
| Manganese (µg/L) | 0.20 | 0.34 | 0.64 | 1.13 | 42.5 | 0.67 | 0.65, 0.70 | 258 (16.0) |
| Molybdenum (µg/L) | 1.00 | 21.4 | 36.5 | 57.8 | 343 | 33.2 | 32.0, 34.5 | 6 (0.37) |
| Plutonium (µg/L) | 0.06 | 0.06 | 0.10 | 0.10 | 2.07 | 0.10 | 0.10, 0.10 | 1143 (71.0) |
| Antimony (µg/L) | 0.10 | 0.16 | 0.20 | 0.34 | 7.12 | 0.24 | 0.23, 0.25 | 605 (37.6) |
| Selenium (µg/L) | 2.00 | 22.1 | 37.6 | 56.6 | 303 | 35.1 | 33.9, 36.3 | 2 (0.12) |
| Thallium (µg/L) | 0.05 | 0.06 | 0.10 | 0.16 | 1.46 | 0.11 | 0.11, 0.11 | 352 (21.9) |
| Uranium (µg/L) | 0.01 | 0.01 | 0.01 | 0.02 | 0.84 | 0.01 | 0.01, 0.01 | 1026 (63.8) |
| Zinc (µg/L) | 22.9 | 253 | 452 | 740 | 8910 | 424 | 407, 441 | 0 |

**Supplemental Table 2. Pearson correlation coefficients (p-values) for natural log-transformed urinary metal concentrations at baseline (n = 1609)**

|  | Tungsten | Arsenic | Cadmium |
| --- | --- | --- | --- |
| Arsenic (µg/L) | 0.51 (<0.001) |  |  |
| Cadmium (µg/L) | 0.16 (<0.001) | 0.35 (<0.001) |  |
| Lead (µg/L) | 0.08 (0.001) | 0.23 (<0.001) | 0.33 (<0.001) |

**Supplemental Table 3. Differences in baseline characteristics among participants missing fasting insulin or fasting glucose at baseline and those not missing fasting insulin or fasting glucose ^a^**

|  | Participants with fasting glucose and fasting insulin  n (%) or mean (standard deviation) | Participants missing either fasting glucose or fasting insulin  n (%) or mean (standard deviation) | p-value |
| --- | --- | --- | --- |
| Total | 1431 (88.9) | 178 (11.1) |  |
| Age (years) | 54.1 (12.1) | 55.1 (13.4) | 0.311 |
| Sex |  |  | 0.587 |
| Men | 674 (47.1) | 80 (44.9) |  |
| Women | 757 (52.9) | 98 (55.1) |  |
| Ethnicity |  |  | <0.001* |
| Hispanic | 651 (45.5) | 122 (68.5) |  |
| Non-Hispanic | 780 (54.5) | 56 (31.5) |  |
| BMI (kg/m^2^) | 26.6 (4.7) | 27.8 (5.3) | 0.001* |
| Urinary W (µg/L) | 0.68 (1.35) | 0.50 (1.04) | 0.083 |

* Significant with p < 0.05

^a^ Differences between baseline categorical and continuous covariate measurements were assessed using chi-squared and two sample t-tests with equal variances, respectively

**Supplemental Table 4. Differences in baseline characteristics of participants by incident diabetes status (n = 1078) ^a^**

|  | Participants that did not develop diabetes  n (%) or mean (standard deviation) | Participants that did develop diabetes  n (%) or mean (standard deviation) | p-value |
| --- | --- | --- | --- |
| Total | 959 (89.0) | 119 (11.0) |  |
| Age | 52.2 (12.5) | 55.2 (10.3) | 0.011* |
| Sex |  |  | 0.594 |
| Men | 460 (48.0) | 54 (45.4) |  |
| Women | 499 (52.0) | 65 (54.6) |  |
| Ethnicity |  |  | <0.001* |
| Hispanic | 376 (39.2) | 73 (61.3) |  |
| Non-Hispanic | 583 (60.8) | 46 (38.7) |  |
| Education |  |  | <0.001* |
| < 12 years | 218 (22.8) | 50 (42.0) |  |
| 12 years | 341 (35.6) | 39 (32.8) |  |
| > 12 years | 398 (41.6) | 30 (25.2) |  |
| Smoking status ^b^ |  |  | 0.336 |
| Never | 426 (44.5) | 58 (48.7) |  |
| Current | 244 (25.5) | 33 (27.7) |  |
| Former | 288 (30.1) | 28 (23.5) |  |
| Hypertension prevalence | 270 (28.2) | 49 (41.2) | 0.003* |
| Body mass index (kg/m^2^) | 25.5 (4.25) | 28.8 (4.29) | <0.001* |
| Caloric intake (kcal/day) ^c^ | 1549 (561) | 1506 (648) | 0.434 |
| Alcohol (g/week) ^c^ | 48.4 (110) | 41.0 (118) | 0.498 |

* Significant with p < 0.05

^a^ Differences between baseline categorical and continuous covariate measurements were assessed using chi-squared and t-tests, respectively, among those who developed diabetes (n = 119) and those who did not (n = 959) among participants without diabetes at baseline (n = 1078).

^b^ Smoking status of never was defined as <100 cigarettes in lifetime. Smoking status of current was defined as ≥100 cigarettes in lifetime and currently a smoker. Smoking status of ever was defined as ≥100 cigarettes in lifetime and not currently a smoker.

^c^ Caloric intake and alcohol intake were measured using a food frequency questionnaire.

**Supplemental Table 5. Cross-sectional associations between tungsten and continuous diabetes measures at baseline stratified by sex and ethnicity ^a^**

|  | **Males ^a^** | **Females** | **Non-Hispanics ^a^** | **Hispanics** |
| --- | --- | --- | --- | --- |
|  | β (95% CI) | β (95% CI) | β (95% CI) | β (95% CI) |
| **Natural log-transformed fasting glucose** | *n = 728* | *n = 839* | *n = 817* | *n = 750* |
| Main model ^b^ | 0.005 (-0.016, 0.026) | -0.002 (-0.023, 0.020) | 0.009 (-0.008, 0.025) | -0.008 (-0.034, 0.018) |
| Further adjusted model ^c^ | -0.012 (-0.037, 0.012) | -0.013 (-0.037, 0.011) | -0.008 (-0.027, 0.011) | -0.019 (-0.048, 0.011) |
| **Natural log-transformed 2-hour glucose** | *n = 690* | *n = 795* | *n = 785* | *n = 700* |
| Main model | 0.022 (-0.013, 0.056) | -0.013 (-0.045, 0.020) | 0.011 (-0.018, 0.040) | 0.000 (-0.039, 0.039) |
| Further adjusted model | -0.014 (-0.053, 0.026) | -0.013 (-0.049, 0.024) | -0.009 (-0.042, 0.023) | -0.008 (-0.052, 0.036) |
| **Natural log-transformed HOMA-IR ^d^** | *n = 654* | *n = 747* | *n = 768* | *n = 633* |
| Main model | 0.075 (0.030, 0.120)* | -0.001 (-0.047, 0.046) | 0.028 (-0.014, 0.070) | 0.068 (0.018, 0.119)* |
| Further adjusted model | 0.046 (-0.007, 0.098) | -0.008 (-0.060, 0.044) | 0.008 (-0.040, 0.056) | 0.056 (-0.001, 0.113) |
| **Natural log-transformed HOMA-β ^d^** | *n = 652* | *n = 747* | *n = 768* | *n = 631* |
| Main model | 0.048 (0.005, 0.091)* | -0.019 (-0.066, 0.028) | 0.003 (-0.035, 0.041) | 0.049 (-0.006, 0.104) |
| Further adjusted model | 0.047 (-0.003, 0.097) | -0.003 (-0.055, 0.050) | 0.012 (-0.032, 0.056) | 0.059 (-0.003, 0.121) |

* Significant with p < 0.05

^a^ Stratified models did not include the variable stratified on as a covariate.

^b^ The main model adjusted for age (years), sex, ethnicity (Hispanic/non-Hispanic), education (<12/12/>12 years), smoking status (current/former/never), hypertension (dichotomous), body mass index (kg/m^2^), caloric intake (kcal/day), alcohol intake (g/week), and urinary creatinine (g/L).

^c^ The further adjusted model adjusted for all covariates in the main model and also natural log-transformed arsenic, cadmium, and lead.

^d^ Homeostatic Model Assessment of Insulin Resistance (HOMA-IR) = [fasting insulin (μU/mL) x fasting glucose (mg/dL)] / 405; Homeostatic Model Assessment of beta cell function (HOMA-β) = [20 x fasting insulin (μU/mL)] / [fasting glucose (mg/dL) - 63]

**Supplemental Table 6. Associations between tungsten and diabetes measures in models excluding participants with tungsten exposure concentrations below the limit of detection**

|  | **Cross-sectional associations** | **Longitudinal associations** |
| --- | --- | --- |
|  | β or OR ^a^ (95% CI) | β or SHR ^a^ (95% CI) |
| **Natural log-transformed fasting glucose** | *n = 1048* | *n = 693* |
| Main model ^b^ | -0.008 (-0.025, 0.009) | 0.006 (-0.001, 0.012) |
| Further adjusted model ^c^ | -0.024 (-0.042, -0.005)* | 0.002 (-0.005, 0.010) |
| **Natural log-transformed 2-hour glucose** | *n = 993* | *n = 688* |
| Main model | -0.002 (-0.028, 0.024) | 0.012 (-0.006, 0.030) |
| Further adjusted model | -0.022 (-0.051, 0.007) | 0.012 (-0.008, 0.033) |
| **Natural log-transformed HOMA-IR ^d^** | *n = 929* | *n = 693* |
| Main model | 0.032 (-0.004, 0.068) | 0.044 (0.018, 0.071)* |
| Further adjusted model | 0.011 (-0.030, 0.051) | 0.034 (0.004, 0.064)* |
| **Natural log-transformed HOMA-β ^d^** | *n = 927* | *n = 693* |
| Main model | 0.023 (-0.013, 0.058) | 0.023 (-0.005, 0.052) |
| Further adjusted model | 0.031 (-0.009, 0.071) | 0.026 (-0.007, 0.058) |
| **Diabetes ^e^** | *n = 1053* | *n = 694* |
| Main model | 0.93 (0.81, 1.07) | 1.24 (1.02, 1.50)* |
| Further adjusted model | 0.84 (0.71, 0.99)* | 1.16 (0.93, 1.45) |

^*^Significant with p < 0.05

^a^ OR = odds ratio; SHR = sub-distribution hazard ratio

^b^ The main model adjusted for age (years; treated as time variable in Fine and Gray competing risks regression models),

sex, ethnicity (Hispanic/non-Hispanic), education (<12/12/>12 years), smoking status (current/former/never), hypertension (dichotomous), body mass index (kg/m^2^), caloric intake (kcal/day), alcohol intake (g/week), and urinary creatinine (g/L).

^c^ The further adjusted model adjusted for all covariates in the main model and also natural log-transformed arsenic, cadmium, and lead.

^d^ Homeostatic Model Assessment of Insulin Resistance (HOMA-IR) = [fasting insulin (μU/mL) x fasting glucose (mg/dL)] / 405; Homeostatic Model Assessment of beta cell function (HOMA-β) = [20 x fasting insulin (μU/mL)] / [fasting glucose (mg/dL) - 63]

^e^ Diabetes measure was prevalence for the baseline analysis and incidence for the prospective analysis.

**Supplemental Table 7. Associations between urinary tungsten and diabetes incidence and prevalence stratified by sex and ethnicity**

|  | **Males ^a^** | **Females** | **Non-Hispanics ^a^** | **Hispanics** |
| --- | --- | --- | --- | --- |
| **Diabetes prevalence; OR ^b^ (95% CI)** | *n = 730* | *n = 843* | *n = 821* | *n = 752* |
| Main model ^c^ | 1.09 (0.92, 1.30) | 0.86 (0.71, 1.04) | 1.09 (0.91, 1.31) | 0.89 (0.74, 1.07) |
| Further adjusted model ^d^ | 0.91 (0.74, 1.12) | 0.82 (0.65, 1.02) | 0.95 (0.77, 1.17) | 0.79 (0.63, 0.99)* |
| **Diabetes incidence; SHR ^b^ (95% CI)** | *n = 501* | *n = 555* | *n = 621* | *n = 435* |
| Main model | 1.37 (1.09, 1.71)* | 1.24 (0.96, 1.61) | 1.43 (1.11, 1.83)* | 1.18 (0.96, 1.45) |
| Further adjusted model | 1.20 (0.90, 1.60) | 1.28 (0.97, 1.69) | 1.32 (1.00, 1.73) | 1.20 (0.95, 1.50) |

^*^Significant with p < 0.05

^a^ Stratified models did not include the variable stratified on as a covariate.

^b^ OR = odds ratio; SHR = sub-distribution hazard ratio

^c^ The main model adjusted for age (years), sex, ethnicity (Hispanic/non-Hispanic), education (<12/12/>12 years), smoking status (current/former/never), hypertension (dichotomous), body mass index (kg/m^2^), caloric intake (kcal/day), alcohol intake (g/week), and urinary creatinine (g/L).

^d^ The further adjusted model adjusted for all covariates in the main model and also natural log-transformed arsenic, cadmium, and lead.

**Supplemental Figure 1. Distribution of urinary tungsten (W) levels at baseline**

**Supplemental Figure 2. Distribution of natural log-transformed urinary tungsten (lnW) at baseline**

**Supplemental Figure 3. Kaplan-Meier survival estimates by urinary W levels above or below median (0.22 µg/L)**

**Supplemental Figure 4. Directed acyclic graph of the association between W and diabetes.** Green arrows represent pathways from the exposure (tungsten and other metals) to the outcome (cardiometabolic disease) or a parent of the outcome. Pink arrows represent confounding pathways. Figure adapted from Riseberg et al. (2020)^39^

**Supplemental Figure 5. Results from the Bayesian kernel machine regression model assessing the cross-sectional association between natural log-transformed metals and diabetes.** Quartiles describe the metal concentrations (for the metal along the y axis). Models were adjusted for age, sex, ethnicity (Hispanic/non-Hispanic), education (<12/12/>12 years), smoking status (never/former/current), hypertension (dichotomous), body mass index (kg/m^2^), caloric intake (kcal/day), alcohol intake (g/week), and urinary creatinine (g/L). (As = arsenic; Cd = cadmium; Cs = cesium; Cu = copper; Mn = manganese; Mo = molybdenum; Pb = lead; Sb = antimony; W = tungsten; Zn = zinc).
